# Biooxidation of Arsenopyrite by *Acidithiobacillus ferriphilus* QBS 3 Exhibits Arsenic Resistance Under Extremely Acidic Bioleaching Conditions

**DOI:** 10.3390/biology14050550

**Published:** 2025-05-15

**Authors:** Run Liu, Siyu Liu, Xiaoxuan Bai, Shiping Liu, Yuandong Liu

**Affiliations:** 1 Hubei Provincial Key Laboratory of Natural Products Research and Development, School of Biology and Pharmacy, Three Gorges University, Yichang 443002, China; 202208600021012@ctgu.edu.cn (R.L.);; 2 Key Laboratory of Biohydrometallurgy of Ministry of Education, School of Minerals Processing and Bioengineering, Central South University, Changsha 410083, China

**Keywords:** biooxidation, arsenopyrite, *Acidithiobacillus ferriphilus*, arsenic resistance leaching mechanism

## Abstract

As arsenopyrite is a typical arsenic-bearing sulfide ore, the biooxidation process of arsenopyrite is of great significance for the extraction of gold from arsenic-bearing gold ores and the generation of arsenic-bearing acid mine drainage. During the biooxidation of arsenopyrite, a large amount of arsenic is produced, which inhibits the growth and metabolism of microorganisms and thus affects the extraction of gold from arsenic-bearing gold ores. Therefore, the screening and enrichment of microorganisms with high arsenic resistance have become important aspects in the study of arsenopyrite biooxidation. As described in this paper, through arsenic acclimation, the maximum arsenic tolerance concentration of *Acidithiobacillus ferriphilus* QBS 3 isolated from arsenic-containing acid mine drainage was increased to 80 mM As(Ⅲ) and 100 mM As(V). Microorganisms with high arsenic resistance showed better bioleaching performance for arsenopyrite. After 18 days of bioleaching, the leaching rate of arsenopyrite reached 100% at a pulp concentration of 0.5%, and after 30 days of bioleaching, the leaching rate of arsenopyrite was 79.96% at a pulp concentration of 1%. Currently, research on arsenopyrite mainly focuses on the control and optimization of environmental conditions, but there have been few studies on the biooxidation process of arsenopyrite at the protein and gene levels. Therefore, combining the results of a one-month bioleaching experiment on arsenopyrite by *A. ferriphilus* QBS 3 and the analysis of arsenic resistance genes, a bioleaching model of arsenopyrite was constructed, which laid an experimental basis and theoretical foundation for improving the gold recovery rate from refractory arsenic-bearing ores and exploring the arsenic resistance mechanism of microorganisms during the arsenopyrite leaching process.

## Simple Summary

The bio-oxidation of arsenopyrite is an important factor resulting in arsenic-polluted acidic mine drainages and has been widely utilized for the bioprocessing of arsenic-bearing gold ores in industry. It is very significant and interesting to explore how the microorganisms thriving in these environments can tolerate the extreme toxicity of high concentration of arsenic while exerting bio-oxidation activities. In this paper, the arsenic resistance features, arsenopyrite leaching effect and arsenic resistance genes of the bioleaching bacterium *Acidithiobacillus ferriphilus* QBS 3 were investigated and a putative arsenic resistance mechanism model of arsenopyrite bio-oxidation was first proposed at the gene level. The high arsenic resistance of this bacterium leads to its excellent capability for arsenopyrite bio-oxidation. The arsenic resistance mechanism results from the synergic actions of the arsenic resistance enzymes of the bacterium and the ferric ions generated by its ferrous oxidation. These results will greatly contribute to the prevention and control of arsenic pollution in environmental protection and the extraction of metals from arsenic-bearing ores in industrial applications.

## 1. Introduction

Acid mine drainage (AMD) poses a significant global environmental challenge due to the biochemical oxidation of metal sulfides in mine wastes, leading to the release of sulfate, protons, and toxic heavy metal ions into the surrounding environment [1,2]. Arsenic, the 20th most abundant element in the Earth’s crust, is a major pollutant in AMD due to its association with sulfide ores such as those of gold, copper, and iron [3]. The weathering of arsenic-bearing sulfide ores releases substantial amounts of arsenic into the environment, with microorganisms playing pivotal roles in the mobilization process [4]. Understanding the bioleaching mechanisms of these sulfides is essential for evaluating arsenic pollution in the environment.

To date, more than 100 types of arsenic-bearing sulfide ores have been identified, with arsenopyrite being the most prevalent and a primary carrier of gold [5]. Researchers often focus on arsenopyrite due to its common occurrence. Biological pre-oxidation has proven effective in reducing the impact of arsenopyrite in arsenic-containing refractory gold ores [6,7]. Therefore, investigating the biological oxidation behavior of arsenopyrite is crucial not only for assessing arsenic release but also for enhancing gold extraction rates from arsenic-containing refractory gold deposits. Previous research has shown that in the biological oxidation of arsenopyrite, the ferric iron (Fe^3^⁺) exhibits a greater tendency to oxidize arsenopyrite than As (Ⅲ), indicating that As predominantly exists as As (Ⅲ) in the leaching solution [8]. Significant levels of As(V)in the solution are typically observed only when pyrite or chalcopyrite is present [9].

Arsenic, a heavy metal, poses a significant threat to microbial growth. The toxicity of arsenic varies depending on its valence states; notably, As (Ⅲ) exhibits 25–60 times greater toxicity than As(V) [10]. Despite this, As(V) is equally harmful. As(Ⅲ) disrupts enzyme function by binding to sulfhydryl and hydroxyl groups on proteins, leading to reduced microbial viability or death [11]. On the other hand, As(V)acting as a phosphate analog, interferes with oxidative phosphorylation, inhibiting microbial metabolic activity [12]. However, this inhibition can be mitigated by the addition of phosphate. In the context of leaching processes, the vitality of microorganisms is crucial in determining the efficiency of arsenopyrite oxidation. Consequently, microorganisms with high arsenic resistance are being considered as industrial strains for pretreating arsenic-containing refractory gold ores [13,14,15]. During the subsequent biological pre-oxidation of arsenic-containing gold ores, these resistant microorganisms exhibit robust vitality, showcasing significant potential for arsenopyrite leaching. Consequently, the primary focus in arsenopyrite bioleaching lies in the screening of microorganisms with high arsenic resistance.

Arsenic acclimation significantly enhances the maximum arsenic tolerance of microorganisms. Leng Feifan et al. demonstrated that the As (Ⅲ) tolerance of *A. ferrooxidans BY-3* increased to 4.74 g/L post acclimation while *A. ferrooxidans TKY-2* exhibited an As(V) tolerance of 8 g/L [16]. Arsenic acclimation also raised *L. ferriphilum*’s tolerance to 7 g/L, boosting leaching efficiency by 10% compared to the original strain [17]. Additionally, *L. ferriphilum YSK* achieved a maximum arsenic tolerance of 4.88 g/L through acclimation. Co-culturing with *A. thiooxidans* increased arsenic tolerance 6-fold, upregulating arsenic resistance genes arsR, arsB, and arsC [18]. Arsenic acclimation effectively enhances microorganisms’ arsenic tolerance and leaching efficiency. Understanding arsenic resistance mechanisms is crucial for improving arsenic tolerance, including arsenic tolerance, oxidation, reduction, and methylation pathways. The arsenic tolerance system, regulated by the arsR transcription factor, is commonly found within the ars operon. This system comprises the transcriptional regulator (arsR), transmembrane pump (arsB), and arsenate reductase (arsC), forming the fundamental arsenic tolerance mechanism [19]. As research on arsenic resistance in leaching bacteria progresses, additional arsenic resistance genes such as arsT and arsD have been identified [20]. The arsenic methylation mechanism, which converts toxic inorganic arsenic into less harmful organic forms, plays a key role in the environmental migration and transformation of arsenic. These mechanisms are prevalent in *A. ferrooxidans* genomes, making them vital in arsenic-containing gold mines due to their robust iron and sulfur metabolism [21]. However, their activity is influenced by arsenic ion concentrations in leachates. Investigating the arsenic resistance mechanisms of this strain is essential for its role in pretreating arsenic-containing gold mines. Although a study compared and analyzed arsenic resistance gene clusters in *A. ferrooxidans*, the expression of these genes during leaching remains unexplored [21]. The arsenic resistance mechanisms of *A. ferrooxidans*, encompassing tolerance, reduction, and methylation, underpin the biological leaching of arsenopyrite by *A. ferrooxidans* [21].

This study investigated *A. ferriphilus*, isolated from arsenic-containing acidic mine wastewater, through arsenic stress adaptation and the subsequent bioleaching of arsenopyrite. Initially classified under *A. ferrooxidans*, *A. ferriphilus* was later identified as a distinct species [22,23]. While the arsenic resistance mechanism of *A. ferriphilus* remains underexplored, extensive research has been conducted on *A. ferrooxidans*. Genome analysis and PCR techniques are frequently employed in such studies [24]. We examined the arsenic resistance mechanism during *A. ferrooxidans* leaching using genome analysis and real-time quantitative reverse transcription PCR (qrt-PCR). This allowed us to model the microbial leaching process at the genetic and protein levels, offering a theoretical and practical framework for managing acid mine waste.

## 2. Materials and Methods

### 2.1. Strain and Culture Conditions

*A. ferriphilus QBS 3* isolated from an acid mine drainage of sulfide minerals containing rich toxic ions of Zn, Pb, As, Cu, etc. in Qibaoshan, Liuyang City, Hunan Province, is stored in the Key Laboratory of Biometally of Central South University. The strain was cultivated in shake flasks under the following conditions: pH 2.0, temperature 30 °C, and rotational speed of 170 r/min. The growth medium, 9 K, was composed of (NH_4_)_2_SO_4_ 3.0 g/L, Na_2_SO_4_ 1.41 g/L, KCl 0.1 g/L, K_2_HPO_4_ 0.05 g/L, MgSO_4_∙7H_2_O 5.0 g/L, Ca(NO_3_)_2_ 0.1 g/L, and FeSO_4_∙7H_2_O 44.7 g/L. The pH of the medium was adjusted to 2.0 with sulfuric acid. All chemical substances in the culture medium originate from China National Pharmaceutical Group Chemical Reagents Co., Ltd., Beijing, China.

### 2.2. Mineral Sample

The mineral samples utilized in this research were sourced from the Metallurgical Laboratory of Central South University. The elemental composition of the minerals was analyzed using X-ray fluorescence spectroscopy (XRF, Axios mAX) (Changsha Powder Research Institute, Changsha, China) and was found to be as follows (wt.%): As, 39.02; Fe, 31.44; S, 15.57. X-ray diffraction (XRD) analysis (Nawei, Japanese Confucianism, Smartlab, Tokyo, Japan) revealed that the mineral predominantly consisted of arsenopyrite (see Figure S1). Subsequent grinding of the mineral through a 200–400 mesh sieve yielded particles ranging from 38 to 75 μm in size. The XRF results are shown in Table 1.

### 2.3. Arsenic Stress Experiment

In this study, As(III) solution was prepared using sodium arsenite while As(V) solution was prepared using sodium hydrogen arsenate heptahydrate. Microorganisms were inoculated into shaking flasks containing As(III) and As(V) at an inoculation density of 1 × 10^9^ cell/mL ferrous iron as the energy source. The strains were acclimated to arsenic using a continuous subculturing method. Upon reaching a stable stage of growth and multiplication, the bacteria were transferred to the next higher concentration gradient (increment at 10 mM intervals). Samples were examined under fluorescence microscopy every 12 h and viable bacteria were counted. When no cells were observed in the microscopic field or the cells stopped moving, arsenic acclimation was considered to be complete.

### 2.4. Bioleaching Experiment

Cultures were conducted in 250 mL Erlenmeyer flasks, each containing 100 mL of sterilized basal medium and either 0.5 g or 1 g of arsenopyrite. The initial pH of the medium was adjusted to pH 2.0 ± 0.05 using 1 M sulfuric acid. A one-month bioleaching experiment was carried out with a cell density of 1 × 10^7^ cells/mL post inoculation. The pH was maintained every three days by dropwise addition of 20 µL sulfuric acid. Incubation was performed in a shaker at 170 rpm and 30 °C. Leaching parameters including pH, redox potential (vs. SCE), free cell density, and arsenic concentration were monitored at regular intervals. Average values from replicate experiments were used for analysis. The weight of each flask was recorded before measurements. Evaporation losses were compensated with pH 2.0 water while sampling volumes were replenished with an equal amount of 9 K basal medium. Solution pH was measured using a PHS-3C (Shanghai Leici, Shanghai, China) pH meter. Redox potential (vs SCE) was determined using a platinum electrode and a Ag/AgCl₂ reference electrode, also using the PHS-3C. Cell density was assessed by direct counting using a hemocytometer. Arsenic concentration was analyzed via ICP-AES (IRIS Intrepid II XSP, Thermo Fisher, Waltham, MA, USA). The arsenic valence during arsenopyrite biooxidation was determined using the arsenic molybdenum blue method [25]. Total Fe and Fe(II) in the leach solution were quantified using the Lingfilolin method [26]. At the conclusion of the leaching experiments, the samples were washed with pH 2.0 water, dried, and sent for XRD analysis. Bacteria collected at the end of the leaching experiment were pretreated following Amanze’s protocol and examined via electron microscopy [27]. Abiotic controls were conducted under identical conditions. All experiments were performed in triplicate. The leaching rate was calculated as follows:

$$\;Leaching\;Rate=\frac{The\;concentration\;of\;arsenic\;ions\;in\;solution}{10\times arsenopyrite\times As(wt.\%)}$$

### 2.5. Bioinformatics Analysis

The genome of the iron and sulfur-oxidizing bacteria QBS 3 from NCBI (accession number: GCA_046254925.1) was obtained for comparative analysis with the BacMet database [28], a repository of experimentally validated heavy metal genes, to identify sequences associated with arsenic resistance. Arsenic resistance gene clusters were selected for mapping using Chiplot (https://www.chiplot.online/) (accessed on 12 January 2025). Experimentally validated arsenic-resistance-related genes from the UniProt database (https://www.uniprot.org/) (accessed on 12 January 2025). were used for comparison with the identified genes. Protein sequence alignment was conducted using MEGA12 [29] for phylogenetic tree construction, MEME (https://meme-suite.org/) (accessed on 12 January 2025), for motif analysis, and the NCBI Conserved Domain Database (https://www.ncbi.nlm.nih.gov/) (accessed on 12 January 2025), for identifying conserved structural domains. The protein sequences were further analyzed and integrated using TBtools-II for bioinformatics analysis.

### 2.6. Total RNA Extraction and qRT-PCR

A 1–5 mL aliquot of logarithmic-phase QBS 3 bacterial culture was centrifuged at 8000× *g* for 5 min at 4 °C to pellet the cells. The pellet was washed twice with pre-chilled PBS (pH 7.4), followed by the addition of 1 mL of TRIzol (Simgen, Hangzhou, China) reagent. The sample was vortexed thoroughly and incubated on ice for 5 min. Subsequently, 200 μL of chloroform was added, and the mixture was shaken vigorously for 15 s, then incubated for 3 min. After centrifugation at 12,000× *g* for 15 min at 4 °C, the upper aqueous phase was carefully transferred to a fresh tube. RNA was precipitated by adding an equal volume of isopropanol and incubating the sample at−20 °C for 10 min. The mixture was centrifuged again at 12,000× *g* for 10 min at 4 °C, the supernatant was discarded, and the RNA pellet was washed twice with 75% ethanol. After being air-dried for 5 min, the pellet was resuspended in 20–50 μL of DEPC-treated water [30]. The RNA concentration, measured using NanoDrop, exceeded 200 ng/μL. Subsequently, 1 μg of total RNA was subjected to reverse transcription and quantitative real-time PCR (qRT-PCR) using the HiScript III RT SuperMix for qPCR (+gDNA wiper) from Novozymes, following the manufacturer’s protocol. The qRT-PCR was performed on Q2000B (Hangzhou Longji Scientific Instrument Company, Hangzhou, China), and the resulting data were used to generate a melting (lysis) curve for analysis. The data were standardized and normalized using the method developed by Kenneth Livak and Thomas Schmittgen in 2001 to calculate the relative expression of the reference gene. The relative expression was then plotted using tbtools [31]. (The primer sequences utilized for qRT-PCR are listed in Table S1).

## 3. Results

### 3.1. Stress Response of QBS 3

**Figure 1:**
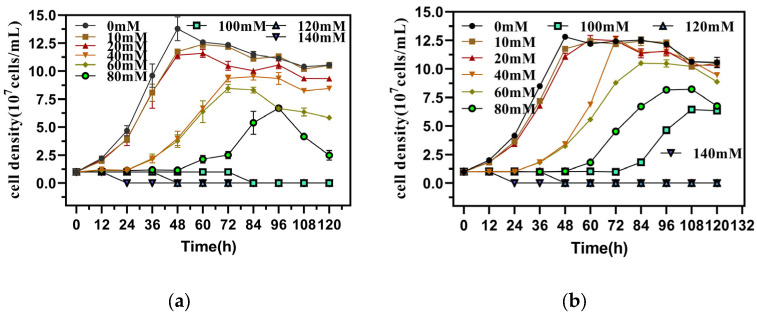
Cell density of QBS 3 under arsenic stress after continuous subculturing: (**a**) cell density under As(Ⅲ) stress; (**b**) cell density under As(V) stress.

The QBS 3 strain, obtained through continuous transfer acclimation, was cultured in media with varying concentrations of sodium arsenite (As(III)) and sodium hydrogen arsenate heptahydrate (As(V)) at an initial cell concentration of 1 × 10^9^ cells/mL. Arsenic acclimation was conducted to sustain elevated metabolic activity under arsenic-induced stress. The growth curve of QBS 3, depicted in Figure 1a,b, exhibited an initial increase, followed by stabilization and subsequent decline. QBS 3 demonstrated a growth rate comparable to arsenic-free conditions when arsenic concentrations were below 20 mM, reaching a peak bacterial density of approximately 1.2 × 10^8^ cells/mL. However, growth rates started to diminish when arsenic concentrations surpassed 60 mM, with an adaptation phase characterized by peak bacterial densities of around 1 × 10^8^ cells/mL. Remarkably, QBS 3 exhibited minimal growth beyond arsenic concentrations of 100 mM for As(III) and 120 mM for As(V), indicating its inability to survive under such extreme conditions. These findings suggest that the arsenic tolerance thresholds for QBS 3 are approximately 80 mM for As(III) and 100 mM for As(V). Notably, during ferrooxidans acclimation, QBS 3 displayed higher tolerance to As(III) compared to *A. ferrooxidans BY-3* and *A. ferrooxidans TKY-2* [16]. Among *A. ferrooxidans* strains, QBS 3 exhibited one of the highest arsenic tolerances. In summary, QBS 3 acquired superior arsenic tolerance through arsenic acclimation compared to most leaching microorganisms.

### 3.2. Bioleaching Performance of QBS 3 on Arsenopyrite

**Figure 2:**
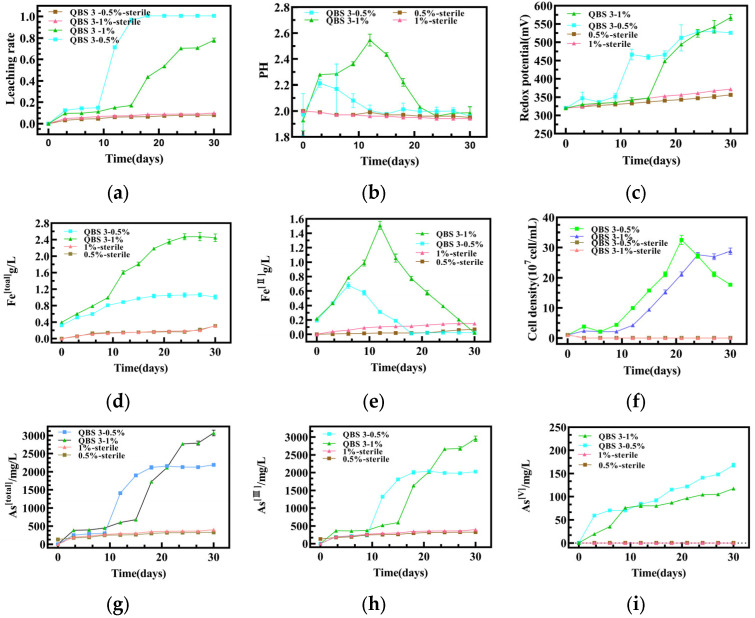
Changes in bioleaching parameters during arsenopyrite oxidation by *A. ferriphilus* QBS 3 (over a 30-day period): (**a**) leaching efficiency, (**b**) pH, (**c**) ORP, (**d**) total iron concentrations, (**e**) ferrous iron concentrations, (**f**) cell density variations during bioleaching, (**g**) total arsenic ion concentration, (**h**) concentrations of As(III), and (**i**) concentrations of As(V).

**Figure 3:**
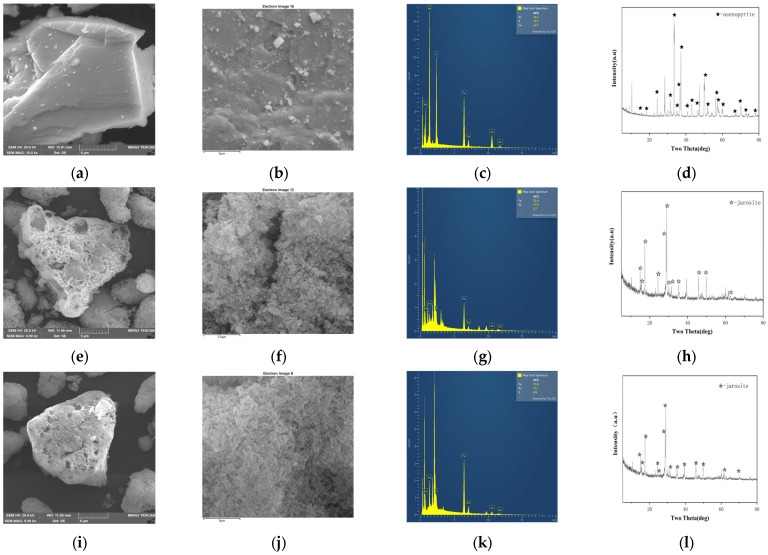
Surface morphology and composition analysis of arsenopyrite slag before and after bioleaching by *Acidithiobacillus ferriphilus* QBS 3: (**a**) SEM image of untreated arsenopyrite surface (smooth, unaltered), (**b**) SEM image of dried, untreated ore slag (no corrosion signs), (**c**) EDS spectrum showing near-1:1:1 Fe:As:S ratio in raw ore, (**d**) XRD pattern of original ore slag, (**e**) SEM image of 0.5% slurry slag (freeze-dried) showing bacterial adhesion and corrosion pits, (**f**) SEM image of 0.5% slurry slag (direct-dried) showing bacterial fragments and material residues, (**g**) EDS of 0.5% slurry slag indicating elemental changes, (**h**) XRD analysis identifying jarosite as the main leaching product, (**i**) SEM image of 1% slurry slag with corrosion fissures and bacterial adhesion, (**j**) SEM image of dried 1% slurry slag with increased bacterial and fragment presence, (**k**) EDS showing slight elemental changes in 1% slurry slag, and (**l**) XRD results confirming jarosite as a predominant passivation product.

The arsenic-tolerant strain QBS 3 was cultured in 9 K medium with 5 g/L and 10 g/L arsenopyrite with an initial cell density of 1 × 10^7^ cells/mL. Over a 30-day bioleaching period, QBS 3 achieved complete leaching (100%) at 5 g/L and 79.92% and leaching at 10 g/L, indicating its high bioleaching efficiency under arsenic stress (Figure 2a). Following domestication, QBS 3 completely oxidized 0.5 g of arsenopyrite within 18 days, resulting in a final arsenic ion concentration of 2.016 g/L (Figure 2g). Subsequently, after 30 days, 1 g of arsenopyrite was leached, leading to a final arsenic ion concentration of 3.125 g/L (Figure 2g). During the initial acclimatization phase (the first 9 days), only minimal changes in arsenic ion concentration were observed. This was likely attributed to low microbial activity and viability, as indicated by stable cell densities during this phase (Figure 2f) [32,33]. An analysis of Figure 2h,i suggests that QBS 3 predominantly oxidized arsenopyrite to As(III), with limited capacity to further oxidize As (III) to As (V). This suggests that As(III), which may be formed as an intermediate during Fe(II) oxidation, accumulates during the leaching process [9]. As QBS 3 adapted to the environment, cell density increased, leading to enhanced arsenopyrite oxidation. Notably, a rapid leaching phase of 5 g/L arsenopyrite was observed between days 9 and 21, aligning with the peak bacterial population in the leaching system. As shown in Figure 3e,f, post-acclimatization bacterial growth exhibited a marked increase, accompanied by fluctuations in ferrous ion concentration. As QBS 3 continued to oxidize arsenopyrite, a gradual decline in total iron concentration in the leachate was detected, potentially forming passivated substances like jarosite or ferric arsenate (Figure 2d,e) [34,35]. The growth of QBS 3 was associated with acid consumption, reflected in pH variations (Figure 2b) during the biooxidation process. These pH variations (Figure 2b) were likely due to the consumption of sulfuric acid during the arsenopyrite oxidation [33,36]. In parallel, steady increase, corresponding to the oxidation of Fe(II) to Fe(III) by QBS 3, served as the primary driver for arsenopyrite oxidation (Figure 2c) [37]. Towards the late stage of biological oxidation, at a 0.5% slurry concentration, complete arsenopyrite oxidation occurred, leading to cell decay and a stabilization of Fe(III) levels. In contrast, at a 1% slurry concentration, incomplete arsenopyrite oxidation was observed, with increasing redox potential indicating ongoing microbial activity. Notably, the oxidation efficiency of QBS 3 declined in the later stages of leaching compared to the initial phase, underscoring the importance of identifying the predominant physical phases of the post-leaching slag in order to improve the overall efficiency of arsenopyrite oxidation.

The SEM images of the bioleached arsenopyrite (Figure 3a–c,e–g,i–k) slag were consistent with Tingting Zhu’s study [35], indicating that compared to the original ore (Figure 3d), jarosite (Figure 3h) may be a key factor contributing to low bioefficiency at the late stage with a 1% slurry concentration (Figure 3l). Jarosite, a prevalent passivation product of arsenopyrite, has been thoroughly examined for its passivation mechanism. During arsenopyrite bioleaching, the rising concentration of iron ions in the solution fosters jarosite formation, aligning with observed leaching outcomes. A particular study identified three primary mechanisms by which jarosite inhibits arsenopyrite leaching: (1) jarosite adsorbs iron and arsenic from a solution, hindering the iron ion oxidation of arsenopyrite and thus decreasing the leaching rate; (2) jarosite formation raises the solution pH, potentially reducing microbial activity by deviating from the optimal pH; and (3) as a dense passivation product, jarosite coats arsenopyrite surfaces, diminishing active sites and impeding the bioleaching process [38,39].

### 3.3. Mining and Analysis of Arsenic Resistance Genes in QBS 3

**Figure 4:**
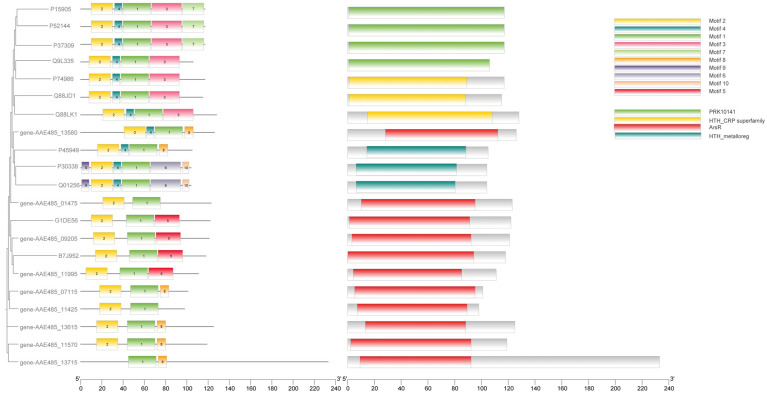
Comparison and analysis of the putative arsR with arsR in the database.

As arsenopyrite oxidized, As(III) was leached from it continuously. The experimental results indicate that at a 10 g/L arsenopyrite concentration, the oxidation rate by A. ferriphilus QBS 3 was limited by the arsenic ion concentration in the leaching solution. Understanding the arsenic resistance mechanisms of QBS 3 is crucial for sustaining oxidative performance under high arsenic levels. Screening the QBS 3 genome (GCA_046254925.1) against the bacmat database identified several arsenic resistance-associated genes (Table 2, Table 3 and Table 4). The transcriptional regulator arsR, present in arsenic and other metal resistance mechanisms such as Cu and Ni, and the ArsR/SmtB family protein cmrT, commonly associated with cadmium (Cd), lead (Pb), and arsenic resistance gene clusters, play key roles. Identifying transcriptional regulators in the arsenic resistance gene cluster is essential for analyzing QBS 3’s arsenic resistance mechanism [40,41]. Protein sequence alignment is vital for studying protein affinity and evolutionary origin. Motif and conserved domain analyses are effective for studying protein functional domains. These analyses, integrated using TBtools-II, facilitated gene family analysis to screen for transcriptional regulators in the arsenic resistance gene cluster (Figure 4).

Comparison with experimentally verified arsR sequences in the UniProt database revealed that genes AAE485_09205, AAE485_11995, AAE485_13580, AAE485_07115, AAE485_11570, and AAE485_13615 exhibit high similarity to the functional and conserved domains of the arsenic resistance regulatory factor arsR. The conserved sequences and structures of arsR suggest shared characteristics with transcription factors involved in other metal-ion resistance mechanisms, underscoring the importance of distinguishing it [40,41].

**Figure 5:**
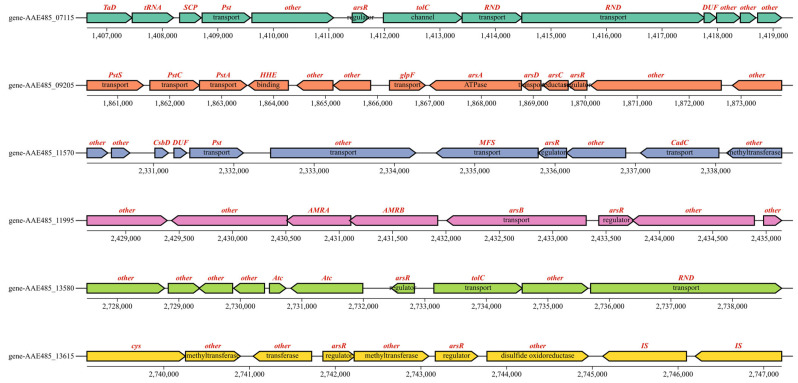
Hypothetical arsR transcription factor and the surrounding arsenic resistance gene cluster.

Bioinformatics analysis around these transcription factors and gene cluster mapping identified potential arsenic resistance gene clusters related to arsR (Figure 5). Gene-AAE485_07115, near the putative phosphate transporter protein gene PSt, forms a complex with outer membrane channel TolC and cation channel protein RND, involved in transporting zinc, cobalt, and lead, which are inefficient at transporting arsenic due to structural mismatches [42]. Genes around gene AAE485_09205 include phosphate transporter and water/glycerol channel proteins and arsenic tolerance system genes arsA, arsC, and arsD. Arsenate is expelled as a phosphate analog via phosphate transporters while arsenite is freely excreted through water/glycerol channels [43]. The ArsA gene encodes an ATPase involved in arsenic efflux and is often co-transcribed with arsB encoding an arsenic pump membrane protein [44]. ArsD facilitates the transport of As(III) into the arsenic resistance system [45]. ArsC is expressed under anaerobic conditions and is responsible for arsenate reduction, converting As(V) to As(III) for efflux through arsB/arsD or water/glycerol channels [45]. The ArsC gene encodes arsenate reductase, which plays a dual role in arsenate reduction and serves as a crucial intermediate in the arsenic methylation pathway [45]. Arsenate reductase converts As(V) to As(III) while arsM facilitates the transformation of As(III) into monomethylarsonic acid (MMA) or dimethylarsinic acid (DMA) [46]. These enzymatic reactions heavily rely on thiol-containing cofactors. The genome harbors arsT, which encodes a thioredoxin-reducing protein.

Thioredoxin-reducing protein (TRX) and glutathione (GSH), both thiol-containing compounds, not only act as electron donors for arsC but also serve as vital cofactors in the methylation process catalyzed by arsM [46]. The manipulator cadC, associated with zinc resistance, is located in the vicinity of gene AAE485_11570. Gene-AAE485_11995, located near the arsenic resistance gene arsB, was found to be associated with genes involved in DNA repair. In the vicinity of gene AAE485_13580, the outer membrane channel protein tolC and the multidrug-resistant channel family protein RND were identified. Based on our analysis, we propose the following putative arsR transcription factors, with gene AAE485_09205, gene AAE485_11995, and gene AAE485_13615 being proposed as candidate arsenic resistance arsR transcription factors.

**Figure 6:**
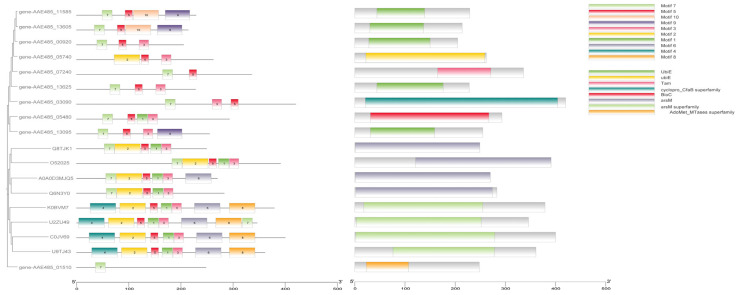
Comparison and analysis of putative arsM with arsM in the database.

The identification of arsenic methylation genes in QBS 3 is crucial for understanding its role in arsenic transport and remediation. The experimentally validated arsM from the UniProt database was compared with putative arsM sequences for phylogenetic tree construction, motif analysis, and conserved structural domain analysis. The putative genes showed distant relationships with the database genes with limited overlap in conserved structural domains (Figure 6). Most bacteria encoded the arsenic methylase protein arsM with multiple conserved cysteine arsenic (III) binding sites, albeit with varying positions among different bacteria. Notably, significant differences in cysteine binding positions were observed between cyanobacteria *Synechocystis* sp. *PCC6803* and sulfate-reducing bacteria *Clostridium* sp. *BXM* [47]. While certain conserved regions exist in the primary structure of arsenic methylase, these regions vary across different bacteria, reflecting interactions with adenosine monophosphate methionine and arsenic binding and methylation processes. Consequently, identifying arsenic methylase poses challenges due to these variations. Bioinformatics analysis revealed multiple cysteines in the protein sequence of gene AAE485_13625, potentially serving as arsenic (III) binding sites, albeit with distinct positions compared to other arsenic methylases. Additionally, genes associated with maintaining the arsenic methylation reduction environment (GST/PNDR) were identified near this gene. GST encodes glutathione S-transferase, involved in arsenate methylation, converting arsenate methyl compounds to arsenate hydrogen methyl compounds [48]. These compounds are subsequently exported from the cell and oxidized to arsenate methyl compounds under aerobic conditions. GST-encoded proteins play a critical role in cellular redox balance, mitigating reactive oxygen species and free radicals induced by arsenic stress [49]. In summary, gene AAE485_13625 emerges as a potential candidate arsenic methylation gene.

### 3.4. q-RTPCR Experiments

**Figure 7:**
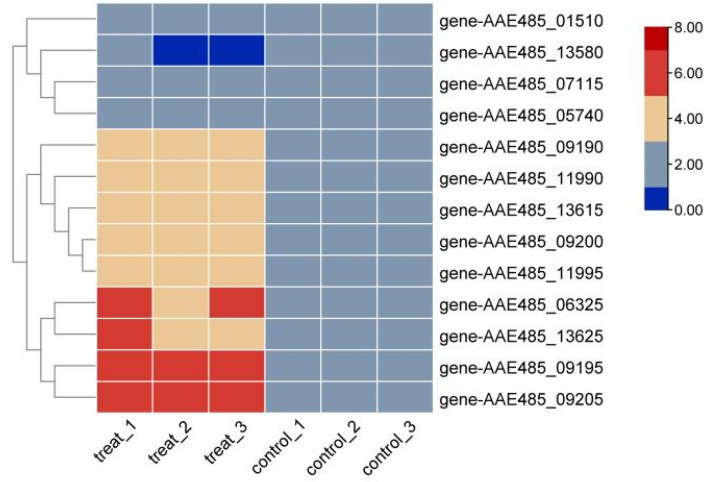
Relative gene expression heatmap.

The putative arsenic resistance genes arsA, arsB, arsC, arsD, arsT, arsR, and arsM in the QBS 3 genome were analyzed using qRT-PCR. QBS 3 cells were subjected to arsenic-free and arsenopyrite bioleaching conditions, followed by RNA extraction for reverse transcription. The resulting cDNA was diluted five-fold and used as template DNA for qRT-PCR. The 16S rRNA gene served as the internal control. Relative gene expression was calculated, normalized for differences between the two conditions, and expressed as base-2 logarithmic fold-changes (Figure 7). Genes AAE 485_06325 (arsT), AAE 485_09190 (arsA), AAE 485_09195 (arsD), AAE 485_09200 (arsC), AAE 485_09205 (arsR), AAE 485_11990 (arsB), AAE 485_11995 (arsR), AAE 485_13615 (arsR), and AAE 485_13625 (arsM) were upregulated under arsenic stress compared to arsenic-free conditions. Other putative arsenic resistance genes showed no significant changes. This suggests that the aforementioned genes are involved in arsenic resistance during arsenopyrite bioleaching while other putative genes are not.

## 4. Discussion

The present study investigated the arsenic acclimation capacity of the strain QBS 3. The results showed that QBS 3 could tolerate up to 80 mM As(III) and 100 mM As(III) after acclimation, enabling it to rapidly adapt to the arsenopyrite bioleaching environment. Following one month of bioleaching, a 0.5% (*w*/*v*) pulp of arsenopyrite was completely oxidized, with an oxidation efficiency of 79.96% for a 1% (*w*/*v*) pulp. The main residual phase after leaching was jarosite, a typical passivation product in arsenopyrite bioleaching. The bioleaching mechanism of QBS 3 involved two-stage acid kinetics, with initial acid consumption followed by acid production, leading to arsenic release and the subsequent formation of arsenic-containing acidic mine drainage (AMD). The oxidation efficiency of arsenopyrite during leaching was limited by the arsenic concentration in the leaching solution and hindered by passivation, suggesting that the use of metal ions, organic acids, or other strategies may be necessary to mitigate passivation effects. Furthermore, genetic studies on the arsenic resistance mechanisms of QBS 3 are needed to better understand its adaptation to high-arsenic environments.

Metallurgical microorganisms exhibit various arsenic resistance mechanisms, including arsenic tolerance, oxidation, arsenate reduction, and arsenic methylation pathways [21]. The genomic analysis of QBS 3 identified potential arsenic resistance genes, such as *arsR* and *arsM*, through bioinformatics screening. The genome revealed genes associated with arsenic resistance (*arsB*, *arsC*, *arsA*, *arsR*, and *arsD*) and potential methylation pathway genes (arsM and arsT). The transcription of arsB, encoding an arsenite channel protein, and arsA, encoding an ATPase subunit, is regulated by the arsR transcription factor binding to As(III) and transported via the *glpF* channel. This process facilitates the transport of As(III) out of the cell to mitigate arsenic stress. The *arsD*-encoded metallochaperone delivers intracellular As(III) to arsenite channel proteins, aiding in the transport of intracellular As(III) to the vicinity of arsenite channel proteins. Under anaerobic conditions, *arsC* encodes arsenate reductase, converting intracellular As(V) to As(III) for external transport through arsB. As(III) serves as substrate for the arsenic methylase *arsM*, which converts toxic arsenite to less harmful organic arsenides. Methylation involves the *arsT* encoded thioredoxin reductase and *GST* encoded glutathione S-transferase, both crucial for arsenic remediation in leaching environments.

To gain insight into the arsenic resistance mechanism of QBS 3 during bioleaching, we conducted qRT-PCR experiments to compare the expression of arsenic resistance genes under leaching conditions with those under arsenic-free conditions. The results showed significant upregulations of gene AAE485_06325 (arsT), gene AAE485_09190 (arsA), gene AAE485_09195 (arsD), gene AAE485_09200 (arsC), gene AAE485_09205 (arsR), gene AAE485_11990 (arsB), gene AAE485_11995 (arsR), gene AAE485_13615 (arsR), and gene AAE485_13625 (arsM) under leaching conditions, suggesting their involvement in arsenic resistance mechanisms during bioleaching. The functions of putative *arsR* and *arsM* genes in bioleaching require further validation in future investigations.

**Figure 8:**
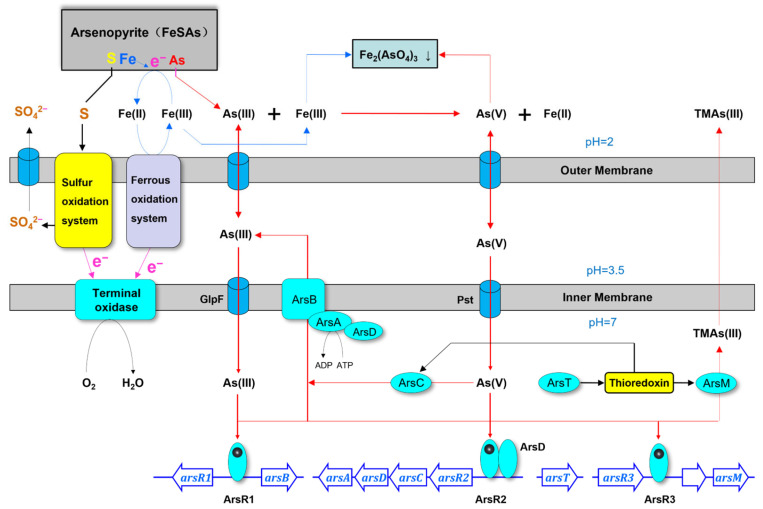
The *QBS 3* bioleaching model for arsenopyrite oxidation.

Based on the genetic (Table 5) and experimental findings discussed above, the theoretical framework of the simulated QBS 3 leaching model (Figure 8) offers insights into enhancing the gold extraction efficiency of arsenic-containing refractory gold ores and addressing the remediation and conversion of arsenic-containing acidic mine wastewater (AMD). Arsenopyrite undergoes oxidation to Fe(II), As (III), and inexpensive sulfides in the presence of H^+^ and O₂. The low-cost sulfide is further oxidized to sulfate by the sulfur oxidation system of the QBS 3 outer membrane and expelled through the surface protein channel. Concurrently, Fe(II) in the leaching solution is oxidized to Fe(III) by the iron oxidation system of the QBS 3 outer membrane. This oxidation process involves electron transfer through iron–sulfur clusters to the terminal oxidase and O₂, yielding water. The As (III) generated from arsenopyrite dissolution enters the periplasmic space through the outer membrane and penetrates the bacteria via aquaglyceroporin channel protein (*glpF*). Within the bacterial system, arsR transcription factors bind to As (III), activating the *ars* gene cluster to mitigate external arsenic stress. Consequently, the arsenic resistance genes arsA/arsB/arsC/arsD/arsT/arsM are upregulated, facilitating the entry of As (III) into the periplasmic space through the arsB-encoded arsenite efflux pump. Here, the ArsA encoded ATPase supplies energy while the *arsD*-encoded metallochaperone aids in binding As (III) to the arsenic channel protein, completing the detoxification process. Additionally, As (III) in the periplasmic space can be extruded from the cell through outer membrane channel proteins. Upon reacting with Fe (III) in the solution, As (III) forms a precipitate of iron arsenate, which is challenging to detect under normal circumstances due to the higher reactivity of Fe (III) towards arsenopyrite oxidation than As (III). As(V) in the leachate enters the periplasmic space and the cell through the phosphate transport system (pst). Subsequently, the arsC-encoded arsenate reductase is activated, converting As(V) to As(III) via the arsenic channel protein in the periplasmic space before excretion. The As (III) produced by arsenate reductase serves as the substrate for arsenic methylation. NADPH donates electrons to thioredoxin disulfide reductase, encoded by arsT, to keep thioredoxin in its reduced form. This reduced thioredoxin serves as an efficient electron donor for arsenic methyltransferase, encoded by arsM, facilitating the methylation of As(III) [45]. This process converts toxic inorganic arsenic into less toxic organic forms, contributing to arsenic remediation and transformation.

## 5. Conclusions

Acclimation to arsenic significantly enhanced the maximum tolerance of QBS 3 to 80 mM As(III) and 100 mM As(V). Post acclimation, QBS 3 demonstrated efficient arsenopyrite oxidation, with the complete oxidation of 0.5 g arsenopyrite occurring within 18 days and an oxidation rate of 79.96% for 1 g arsenopyrite within 30 days. X-ray diffraction (XRD) analysis revealed that the predominant passivation product of the arsenopyrite residue oxidized by QBS 3 was jarosite. Through bioinformatics analysis, the arsenic-resistant gene cluster in the QBS 3 genome was investigated, identifying key genes such as arsR and arsM through quantitative real-time polymerase chain reaction (qRT-PCR). These genes are implicated in the bioleaching behavior of QBS 3, crucial for maintaining high activity under elevated arsenic levels. While these putative arsenic-resistant genes are pivotal in arsenic transformation and repair in bacteria, their functions necessitate further experimental validation. In addition, during the arsenic adaptation process, the complexes or precipitates between ferrous and ferric ions and arsenic ions will change the arsenic ion concentration in the solution, thereby reducing the direct toxicity of arsenic ions to cells. Building upon these findings, a model elucidating the arsenopyrite leaching process by QBS 3 was developed. This model serves as a foundational framework for enhancing gold extraction rates from arsenic-laden refractory gold ore and for arsenic remediation and transformation in acidic wastewater containing arsenic.

## Figures and Tables

**Table 1:** Summary of XRF results for arsenopyrite.

elements	Fe	As	S	O	Zn	Si	Mg
(wt.%)	31.44	39.02	15.57	5.9	2.243	1.83	1.66

**Table 2:** Identification of the putative genes involved in arsenic resistance from *A. ferriphilus* QBS 3.

Name	Gene Locus	Length (AA)	Dir.	Annotation	BACMAT (%)
ArsT	06325	322	↑	Thioredoxin disulfide reductase	42.759
ArsA	09190	589	↑	Arsenical pump-driving ATPase	68.836
ArsD	09195	121	↑	Arsenical resistance operon trans-acting repressor, metallochaperone	59
ArsC	09200	162	↑	Arsenate reductase	36.029
ArsR2	09205	121	↑	Helix-turn-helix domain-containing protein	
ArsB	11990	436	↑	Arsenical pump membrane protein, 11 transmembrane helixes	61.098
ArsR1	11995	120	↓	Helix-turn-helix domain-containing protein(down gene overlap 17 bp)	
ArsR3	13615	124	↓	Metalloregulator ArsR/SmtB family transcription factor	
ArsM	13625	227	↓	Class I SAM-dependent methyltransferase	36.842

In the Table 2, the direction represented by ‘dir’ indicates the direction of the gene, and ‘bacmat (%)’ represents the similarity of the genes in the QBS 3 genome to the corresponding genes in the database.

**Table 3:** Identification of the ArsR genes involved in arsenic resistance from *A. ferriphilus* QBS 3.

Name	Gene Locus	Length (AA)	Dir.	Annotation	BACMAT (%)	True
ArsR	01475	122	↑	Helix-turn-helix transcriptional regulator	42.857	×
CzcR	07115	100	↓	Metalloregulator ArsR/SmtB family transcription factor	45.455	×
ArsR2	09205	121	↑	Helix-turn-helix domain-containing protein		√
ArsR	11425	97	↓	Metalloregulator ArsR/SmtB family transcription factor	34.848	×
ArsR	11570	118	↑	Metalloregulator ArsR/SmtB family transcription factor	33.333	×
ArsR1	11995	120	↓	Helix-turn-helix domain-containing protein		√
CzcR	13580	125	↓	Metalloregulator ArsR/SmtB family transcription factor	46.377	×
ArsR3	13615	124	↓	Metalloregulator ArsR/SmtB family transcription factor		√
ArsR	13715	232	↓	Winged helix-turn-helix domain-containing protein		×

In the Table 3, the direction represented by ‘dir’ indicates the direction of the gene, and ‘bacmat (%)’ represents the similarity of the genes in the QBS 3 genome to the corresponding genes in the database. The symbols indicate the screening and comparison of genes that perform the same function, where √ signifies a candidate gene, and × indicates a gene that cannot be considered a candidate.

**Table 4:** Identification of the ArsM genes involved in arsenic resistance from *A. ferriphilus* QBS 3.

Name	Gene Locus	Length (AA)	Dir.	Annotation	BACMAT (%)	True
ArsM	00920	204	↓	S-adenosylmethionine-dependent methyltransferase activity	27.48	×
ArsM	01510	248	↑	Class I SAM-dependent methyltransferase		×
ArsM	03090	420	↓	Class I SAM-dependent methyltransferase		×
ArsM	05480	292	↓	Malonyl-CoA methyltransferase activity [Evidence IEA]	36.28	×
ArsM	05740	261	↑	Methyltransferase activity [Evidence IEA]	37.03	×
ArsM	07240	336	↑	Class I SAM-dependent methyltransferase		×
ArsM	11585	228	↑	Class I SAM-dependent methyltransferase	25.87	×
ArsM	13095	254	↓	Class I SAM-dependent methyltransferase	33.33	×
ArsM	13605	213	↓	Class I SAM-dependent methyltransferase	31.19	×
ArsM	13625	227	↓	Class I SAM-dependent methyltransferase	36.84	√

In the Table 4, the direction represented by ‘dir’ indicates the direction of the gene, and ‘bacmat (%)’ represents the similarity of the genes in the QBS 3 genome to the corresponding genes in the database.The symbols indicate the screening and comparison of genes that perform the same function, where √ signifies a candidate gene, and × indicates a gene that cannot be considered a candidate.

**Table 5:** Summary of putative QBS 3 arsenic resistance genes.

Name	Gene Locus	Length (AA)	Dir.	Annotation
ArsT	06325	322	↑	Thioredoxin disulfide reductase
ArsA	09190	589	↑	Arsenical pump-driving ATPase
ArsD	09195	121	↑	Arsenical resistance operon trans-acting repressor, metallochaperone
ArsC	09200	162	↑	Arsenate reductase
ArsR_2_	09205	121	↑	Helix-turn-helix domain-containing protein
ArsB	11990	436	↑	Arsenical pump membrane protein, 11 transmembrane helixes
ArsR1	11995	120	↓	Helix-turn-helix domain-containing protein
ArsR_3_	13615	124	↓	Metalloregulator ArsR/SmtB family transcription factor
ArsM	13625	227	↓	Class I SAM-dependent methyltransferase

In the Table 5, the direction represented by ‘dir’ indicates the direction of the gene, and ‘bacmat (%)’ represents the similarity of the genes in the QBS 3 genome to the corresponding genes in the database.

## Data Availability

The data presented in this study are available on request from the corresponding author.

## Supplementary Materials

The following supporting information can be downloaded at: https://www.mdpi.com/article/10.3390/biology14050550/s1, Figure S1. XRD of arsenopyrite; Table S1. Primers use names as well as sequences.
