# Supplementary material for: Biooxidation of Arsenopyrite by Acidithiobacillus ferriphilus QBS 3 Exhibits Arsenic Resistance Under Extremely Acidic Bioleaching Conditions
Source: Biology (Basel). 2025 May 15;14(5):550. doi: 10.3390/biology14050550 (PMC12108572; doi:10.3390/biology14050550)
Supplement: Supplementary file 1 [file biology-14-00550-s001.zip › biology-3562067-supplementary.pdf]

Supplementary material

Biooxidation of Arsenopyrite by *Acidithiobacillus ferriphilus* QBS 3 Exhibits Arsenic Resistance Under Extremely Acidic Bioleaching Conditions

Run Liu [1,2], Siyu Liu [1], Xiaoxuan Bai [1], Shiping Liu [1,*] and Yuandong Liu [2,*]

[1] Hubei Provincial Key Laboratory of Natural Products Research and Development, School of Biology and Pharmacy, Three Gorges University, Yichang 443002, China
[2] Key Laboratory of Biohydrometallurgy of Ministry of Education, School of Minerals Processing and Bioengineering, Central South University, Changsha 410083, China
* Correspondence: yuandong_liu@csu.edu.cn (Y.L.); liuspain@ctgu.edu.cn (S.L.)

Fig S1.XRD of arsenopyrite

XRF analysis (**Fig.**S1) indicates the arsenopyrite sample comprises 39.02% As, 31.44% Fe, and 15.57% S by weight. Fig.S1 presents the XRD pattern of the arsenopyrite sample, revealing a purity exceeding 85%. XRD analysis identifies ZnS and amphibole as impurity phases, with minor components constituting less than 4%. Consequently, the impact of impurities is negligible, affirming arsenopyrite as the predominant phase.

Fig tabe S1.Primers use names as well as sequences

Primers used herein are tabulated as follows.

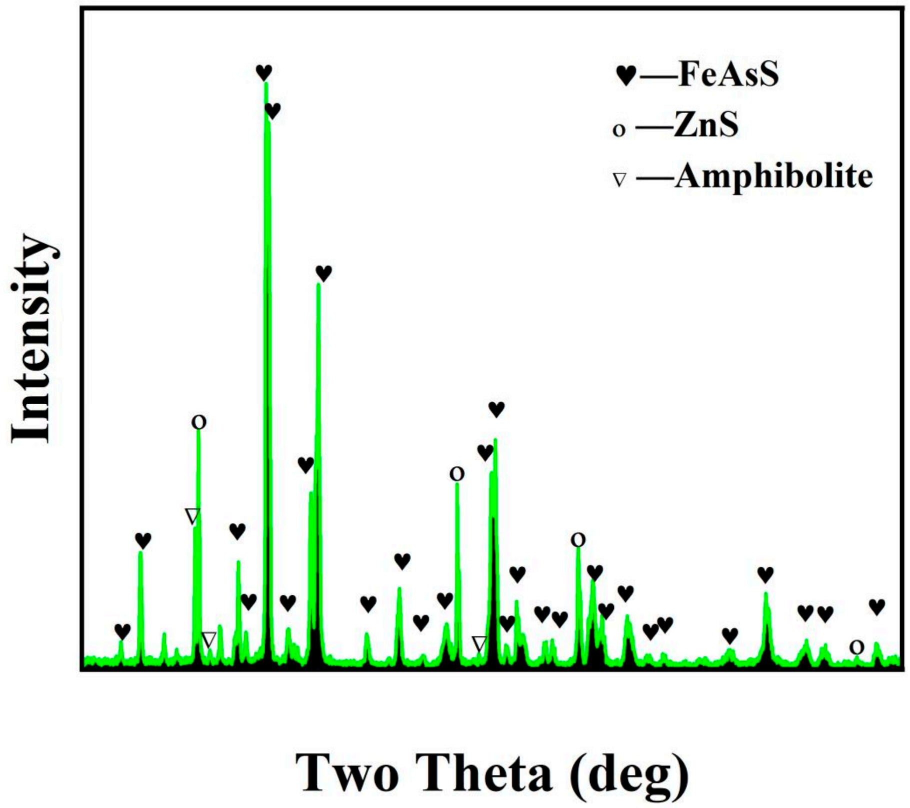

Figure S1.XRD of arsenopyrite

Table S1.Primers use names as well as sequences

name	Primer sequnence
16SF	GCCCTGGACATAAAGGCCAT
16SR	TTGTCCTTAGTTGCCAGCGGTTC
6325F	CTGGGATCACTCTGTGGACG
6325R	TAGCCCGCTTCGTCCATTTT
9190F	CATCCGTCTGGTTCGCTTGG
9190R	CGCTCCCGGTACTCTCGTG
9195F	GTTTTTGGATCGCTCTGGCG
9195R	AGTTCCTCACGGGTCGGATA
9200F	TTCCTGCCGTTCCATCCTTG
9200R	ATGACGATGTCCGGTGTTCC
9205F	GGAAATGCGCCAAGATCACG
9205R	CTCCACCAGCAGGCGATAAA
11990F	TGGTGCTCTGGATCGCATTT
11990R	CTGCGCGGTCACAAAATAGG
11995F	TGCCCGTAGTCTGGACCTTA
11995R	CGTTTGGCACTGTCTGCATT
13615F	ACCAATGTGTCCTTCCATTTGC
13615R	GATTGCAGGATCGCCAACAAC
13625F	TCACCCGCGAAGAATACCAG
13625R	GACACCCAAAGCCCTGATGA
13580F	CTGCCGTATCCGTTGCTTTG
13580R	CTCGAAAACACGGGCGAATC
07115F	ACGCTCCCTGAAAGCTATGG
07115R	GCGAGGTGCTGAGAGATGTT
1510F	GCGCAATGTCTTGTCCTTGG
1510R	TCGACTCCGTGAGAGCCTAT
5740F	GGTGTCGAGTTCGTAGAGGC
5740R	GCGTTCTGGATGGGTCATGT
